# Natural Attenuated Vaccines for *Pasteurella multocida*

**DOI:** 10.3390/biology14111466

**Published:** 2025-10-22

**Authors:** Qingyuan Xu, Lijun Guan, Yun Xue, Zhanqin Zhao

**Affiliations:** 1 Key Laboratory of Animal Bacterial Infectious Disease Prevention and Control Technology, College of Animal Science and Technology, Henan University of Science and Technology, Luoyang 471003, China; w17396352359@126.com (Q.X.); gljguanlijun@163.com (L.G.); xueyun6688@163.com (Y.X.); 2 Key-Disciplines Laboratory of Safety of Environment and Animal Product, College of Animal Science and Technology, Henan University of Science and Technology, Luoyang 471003, China

**Keywords:** *Pasteurella multocida*, pasteurellosis, natural attenuated vaccines, research progress

## Abstract

**Simple Summary:**

*Pasteurella multocida* is a widespread bacterium that causes severe diseases in livestock, leading to substantial economic losses. However, the prevention and control technologies for animal pasteurellosis remain relatively under-developed, with a heavy reliance on the traditional inactivated vaccines and live attenuated vaccines developed in the mid-20th century, i.e., traditional inactivated vaccines, which often provide only limited and short-lived protection. In contrast, live attenuated vaccines can induce broader and longer-lasting immunity. This review focuses on the foundational class of live attenuated vaccines, i.e., the natural attenuated strains, and traces their historical development and application. We synthesize past research to extract key lessons, with the aim of providing theoretical foundations and novel perspectives for the development of a new generation of live-attenuated vaccines against pasteurellosis in livestock and poultry.

**Abstract:**

*Pasteurella multocida* is capable of infecting various animal species, causing hemorrhagic septicemia or infectious pneumonia, and it is widely prevalent and pathogenic worldwide. However, the prevention and control technologies for animal pasteurellosis remain relatively under-developed, with a heavy reliance on the traditional inactivated vaccines and live attenuated vaccines developed in the mid-20th century. A previous systematic review summarized research progress in the field of inactivated vaccines for animal pasteurellosis and revealed that inactivated vaccines exhibit high levels of homologous protective efficacy, albeit with a relatively short duration of immunity and limited cross-protection between different serotypes. Live attenuated vaccines are known to exhibit significant advantages, including a prolonged duration of immunity, strong cross-protective capacity, and low production costs. Throughout the history of vaccine development, various live attenuated vaccines have been extensively used as critical tools for preventing and controlling infectious diseases in animals and humans and combating antimicrobial resistance, substantially reducing the morbidity and mortality rates of regional or international epidemics. The developmental history of live attenuated vaccines against *Pasteurella multocida* has primarily encompassed three distinct research and application periods, characterized by natural attenuated vaccines, traditional attenuated vaccines, and genetically engineered live attenuated vaccines. Here, we comprehensively review the research and application history of natural attenuated vaccines for *Pasteurella multocida*. Our aim was to provide theoretical foundations and novel perspectives for the development of a new generation of live attenuated vaccines against pasteurellosis in livestock and poultry.

## 1. Introduction

*Pasteurella multocida* (*P. multocida*), a Gram-negative coccobacillus belonging to the genus *Pasteurella* within the family Pasteurellaceae, has been identified as a significant pathogen capable of infecting multiple animal species and inducing hemorrhagic septicemia or infectious pneumonia. This organism is distributed worldwide and recognized as an important etiological agent for bacterial zoonoses, with human infections being transmitted via canine or feline bites, scratches, or licking [1,2,3]. Based on capsular antigens, serological classification has established five distinct serogroups (A, B, D, E, F) [4], while lipopolysaccharide (LPS) typing has differentiated 16 serovars (designated 1–16) [4]. Furthermore, disease manifestations, host specificity, pathogenicity, and epidemiological patterns have been demonstrated to correlate significantly with specific capsular-LPS serotype combinations [1,5,6], with outbreaks of avian cholera predominantly associated with A:L1 and A:L3 strains, bovine hemorrhagic septicemia primarily with B:L2 strains, bovine respiratory disease complex with A:L3 serotypes, porcine pneumonic pasteurellosis with D:L6, A:L3 and A:L6 strains (with occasional isolation of F:L3 and B:L2 strains), and rabbit pasteurellosis principally attributed to A:L3 and A:L6 serotypes [7,8,9]. Although the extensive application of antibiotics was previously able to control bacterial diseases in animals, it has also resulted in severe problems, including drug resistance, food safety hazards, and public health challenges [10,11]. Over recent years, policies aimed at reducing the use of antibiotics in animal production have been progressively implemented across multiple nations, imposing more stringent requirements for disease prevention and control measures [12,13]. Consequently, immunization remains the principal strategy for the prevention and control of bacterial infectious diseases in animals.

Globally, existing prevention and control technologies for animal pasteurellosis remain relatively underdeveloped with heavy reliance on traditional inactivated vaccines and live attenuated vaccines developed in the mid-20th century. Although traditional inactivated vaccines exhibit high homologous protective efficacy, they are associated with several limitations including a short duration of immunity, mismatch with currently prevalent serotypes, and weak cross-protection [14]. Live attenuated vaccines are generally associated with significant advantages such as a prolonged duration of immunity, strong cross-protection, and low production costs [15] and primarily comprise natural attenuated vaccines, traditional attenuated vaccines, and genetically engineered attenuated vaccines. During the history of vaccine development for both humans and animals, various live attenuated vaccines, including the LaSota strain of Newcastle disease vaccine [16] and the CU strain of avian pasteurellosis vaccine [17], as well as traditional attenuated vaccines such as the BCG strain of Mycobacterium bovis [18], the GC42 strain of swine erysipelas vaccine [19], and the EO630 strain of the swine pasteurellosis vaccine, have been extensively employed as crucial tools for preventing and controlling infectious diseases and combating antimicrobial resistance, significantly reducing the morbidity and mortality rates of regional and global epidemics [20,21,22]. The live attenuated vaccines against animal pasteurellosis that are commonly used at present include the natural attenuated CU strain and traditional attenuated M-9 and PM-1 strains for avian pasteurellosis in North America and Europe, as well as the traditional attenuated EO630 strain for swine pasteurellosis and G19E40 strain for avian pasteurellosis that is widely utilized in China. Furthermore, certain live attenuated vaccines for animal pasteurellosis, such as the B26-T1200 strain for avian pasteurellosis and CA strain for swine pasteurellosis, have been discontinued due to issues related to weak protective efficacy and poor safety [23,24].

The research and application history of live attenuated vaccines against *Pasteurella multocida* has predominantly included three aspects: natural attenuated vaccines, traditional attenuated vaccines, and genetically engineered attenuated vaccines. Here, we performed a systematic retrospective analysis and prospective evaluation of natural attenuated *Pasteurella multocida* vaccines, with subsequent comprehensive reviews planned for traditional attenuated and genetically engineered attenuated vaccines. Our aim is to provide theoretical foundations and novel perspectives for the research and development of new generation live attenuated vaccines against animal pasteurellosis.

## 2. *Pasteurella multocida* CU Strain Natural Attenuated Vaccine

In 1970, Bierer et al. isolated an attenuated strain of *Pasteurella multocida* from cases of fowl cholera in turkeys that was initially designated as strain CS-148 for identification purposes [25] and subsequently renamed as the CU strain (Clemson University), exhibiting serotype A:3,4. This strain was developed into a commercial vaccine via research conducted by Derieux et al. and has been widely used in several countries, including the United States and Peru [17].

### 2.1. The Influence of Inoculation Routes on the Immunogenic Efficacy of the CU Strain Vaccine

In 1973, Bierer et al. administered the CU strain to turkeys via eight different routes (including drinking water) at varying doses (4.0 × 10^6^–3.0 × 10^8^ CFU/bird), followed by challenge with the P-1059 strain via drinking water (DW) after two weeks. Results demonstrated a survival rate of 30% (6/20) in the control group, while protection rates in the eight vaccinated groups were as follows: drinking water (95%; 19/20), intradermal (85%; 17/20), oral (80%; 16/20), feather follicle (80%; 16/20), ocular (75%; 15/20), palatine cleft (60%; 12/20), cloacal (55%; 11/20), and nasal (35%; 7/20) [26]. In a supplementary experiment, turkeys vaccinated via wing-web puncture (WW) exhibited a protection rate of 97% (39/40), compared to a survival rate of 45% (18/40) in the control group. However, after vaccination through this route, caseous nodules were observed at the inoculation site without other significant clinical manifestations [26]. Collectively, these findings indicate that vaccination routes, including drinking water, intradermal, oral, feather follicle, and WW puncture provided satisfactory levels of protection, whereas ocular, palatine cleft (PC), cloacal, and nasal routes were associated with lower efficacy (Table 1). In 1986, Schlink et al. reported similar experiments demonstrating effective levels of protection following single-dose CU strain vaccination via six different routes: intratracheal injection, bursal perfusion, WW puncture, crop injection, subcutaneous injection (SC), and drinking water (Table 1). Of these, the drinking water and SC injection routes exhibited better safety profiles when compared to the other methods, while the WW puncture vaccination induced the formation of caseous abscesses at the site of inoculation, which eventually developed into scab formation covering the wing area as the disease progressed [27].

In 1980, Derieux et al. inoculated broiler breeder chickens with the CU strain via WW puncture and subcutaneous injection routes (two vaccinations at 14 and 22 weeks of age, 5 × 10^6^ CFU/dose), resulting in 100% survival of immunized birds. When challenged with the X-73 strain through the palatine cleft at 30 weeks of age, protection rates were 87.50% (35/40) and 100% (15/15) for WW puncture and subcutaneous routes, respectively, compared to 3.85% (1/26) survival in the control group. Furthermore, following three vaccinations with the CU strain (at 14, 22, and 34 weeks) and a challenge with the X-73 strain at 68 weeks, protection rates reached 94.44% (19/20) for WW puncture and 100% (17/17) for SC injection compared to 14.71% (6/41) survival in the control groups [28]. These results demonstrated a higher protective efficacy for the two SC CU strain vaccinations when compared to WW puncture administration. In 1982, Cai et al. performed similar experiments and confirmed that SC immunization with the CU strain provided a stronger immune protection than the administration via the drinking water [29] (Table 1).

In 1983, Ghazikhanian et al. used a combined immunization protocol using drinking water (approximately 7.5 × 10^8^ CFU/bird) and WW puncture (approximately 7.5 × 10^7^ CFU/bird) routes with the CU strain in turkeys. Complete survival and the absence of clinical symptoms were observed when primary or booster immunization was administered via the drinking water route. However, when WW puncture booster immunization was conducted following the immunization of primary drinking water, significant clinical manifestations were induced in turkeys, including lameness and wing scabbing. Notably, no clinical symptoms, such as lameness, were observed when WW puncture booster immunization was preceded by three rounds of drinking water immunization. These results indicated the greater safety of the drinking water route when compared to WW puncture inoculation and demonstrated that a prior drinking water immunization could mitigate the adverse reactions caused by a subsequent WW puncture vaccination [30] (Table 1). In 1986, Schlink et al. reported that auditory tube (AT) inoculation frequently led to a reduction in egg production and clinical manifestations such as torticollis in vaccinated chickens, while the immunization via drinking water exhibited both greater safety and protective efficacy when compared to injection into the AT [31] (Table 1).

Based on the safety evaluation results of different administration routes, researchers demonstrated that immunization via the drinking water and SC injection were the safest vaccination routes when chickens were immunized with the CU strain at the same dosage, while AT inoculation, WW puncture, crop injection, intratracheal injection, and bursal injection exhibited relatively lower safety profiles. Notably, AT inoculation was frequently associated with reduced egg production and clinical manifestations such as torticollis in vaccinated chickens [31]. WW puncture inoculation often resulted in the formation of caseous abscesses or scabs at the injection site [26,27,30]. The analysis of protective efficacy data across various administration routes (Table 1) revealed that single-dose immunization with the CU strain via multiple approaches, including drinking water, SC injection, WW puncture, intratracheal injection, bursal injection, and crop injection, could induce satisfactory protective immunity. Therefore, considering both safety parameters and practical applicability in clinical settings, the optimal vaccination strategies for the CU strain were determined to be conventional mass administration methods suitable for intensive poultry farming systems, particularly drinking water, SC injection, and WW puncture.

### 2.2. The Influence of Inoculation Dosage and Frequency on the Immunization Efficacy of the CU Strain Vaccine

In 1977, Coates et al. administered varying doses of the CU strain (4.8 × 10^9^, 6.0 × 10^7^, and 4.0 × 10^5^ CFU/bird via drinking water for 5 h) to 10 weeks of age female Large White turkeys. Except for two deaths (2/30) in the high-dose group, all birds in the other groups survived. After 21 days, a challenge was performed using approximately 100 LD50 of the virulent *Pasteurella multocida* strain P-1059 (serotype A:3) via drinking water (2.0 × 10^7^ CFU/mL for 2 d), with protection rates of 96.4% (27/28), 76.6% (23/30), and 0% (0/30) observed, respectively [32]. These results identified a positive correlation between the protective efficacy of the CU strain and inoculation dose. In 1986, Schlink and Olson confirmed this conclusion by performing WW puncture or SC inoculation in turkeys [27]. When single immunization was performed, the protective efficacy of the CU strain exhibited a positive correlation with the inoculation dose within a certain range, regardless of whether administered via WW puncture or SC injection. However, the WW puncture route induced stronger protective efficacy than SC inoculation (Table 2).

In 1979, Rice et al. administered two immunizations of the CU strain (Group A: 1.5 × 10^8^, 1.5 × 10^8^ CFU/bird; Group B: approximately 1.4 × 10^8^, 3.6 × 10^8^ CFU/bird) to Ross-Arbor Acre broilers via drinking water, followed by challenge with the A:3 type P-1059 strain via the same route. The survival rates were determined to be 50% (10/20) in Group A and 40% (8/20) in the control group, demonstrating no significant protection, whereas Group B showed 75% (30/40) survival compared to 35% (14/40) in the control group indicating protective efficacy. Similar results were obtained when both immunized groups were challenged with the A:1 type X-73 strain [33]. These findings suggested that an increased dosage of secondary immunization enhanced protective efficacy in immunized broilers. In 1980 and 1986, Derieux et al. [28] and Schlink and Olson [31], respectively, reached comparable conclusions by performing immunization-protection experiments employing varying vaccination frequencies and dosages, demonstrating that two or three immunizations conferred stronger protection in turkeys (Table 2). Furthermore, when the combination of drinking water (primary) and AT perfusion (secondary) immunization was implemented, satisfactory protection was achieved even with lower immunization doses, with a protection rate ranging from 70.6% (12/17) to 100% (24/24) (Table 2).

In 1979, Olson administered the CU strain (4 × 10^8^ CFU/bird for two days via drinking water) to 7 weeks of age female Nicholas broad-breasted white turkeys, followed by challenge at 10 weeks of age with wild-type strain 9481, 8579, and 443 (6.0 × 10^9^ CFU/bird via drinking water). Post-challenge survival rates were 24/24 (vaccinated) versus 6/24 (control) for strain 9481; similar results were obtained with strain 8579, while no protection was observed against strain 443 (18/24 survival in both groups) [34]. These findings demonstrated significant variation in the protective efficacy of the CU strain against different clinical strains. Concurrently, Olson reported 91.7–100% protection rates when four different immunization doses (1.6 × 10^10^, 8 × 10^9^, 4 × 10^9^, and 2 × 10^9^ CFU/bird) were administered via drinking water [34] (Table 2). In 1982, Cai et al. conducted similar experiments evaluating various doses of the CU strain (6.7 × 10^7^, 2.5 × 10^7^, 5 × 10^6^, and 2.5 × 10^6^ CFU/bird) in 11 weeks of age Leghorn chickens. Following intramuscular (IM) challenge with strain C48-1 (14 CFU/bird, A:1), all vaccinated groups demonstrated satisfactory levels of protective efficacy, with a protection rate ranging from 75.0% (3/4) to 100% (4/4) [29], thus indicating that SC administration of the CU strain in chickens exhibited a good safety profile and that even lower immunization doses could provide adequate protection (Table 2).

In summary, the CU strain vaccine has been demonstrated to provide protective efficacy in immunized chickens and turkeys against challenge with multiple strain of *Pasteurella multocida*, although significant variations in protection levels were observed against different clinical strain (Table 2). Furthermore, within a certain range, the immunoprotective effect of the CU strain was positively correlated with inoculation dose, while excessive immunization doses were found to induce morbidity and mortality in chickens. A two-dose vaccination regimen with the CU strain was shown to elicit significantly greater protective effects when compared to a single-dose administration, with even lower immunization doses providing satisfactory protection (Table 2).

### 2.3. The Influence of Vaccination Age on the Immunization Efficacy of the CU Strain Vaccine

In 1975, Bierer et al. administered the CU strain (2 × 10^7^ CFU/mL) via drinking water to six groups of day-old turkeys at 1, 5, 10, 15, 20 and 25 days of age, respectively, with protection rates of 2/10, 4/10, 7/10, 9/10, 10/10 and 10/10 observed following challenge with the P-1059 strain at 35 days of age (5 weeks), while the survival rate in the control group was 4/20. When challenged at 12 weeks of age, survival rate of 60% (6/10) and 80% (8/10) were obtained in the 20- and 25-day immunization groups, respectively, whereas lower protection rates ranging from 1/9 to 2/10 were observed in other immunization groups and the survival rate in the control group was 0/19 [17]. These results demonstrated that within a certain age range of chicks, the protective efficacy of drinking water vaccination with the CU strain increases with advancing age (Table 3).

In 1986, Schlink et al. inoculated five groups of 6 weeks of age broad-breasted white turkeys via the WW puncture route with the CU strain (4 × 10^8^ CFU/bird) at 7, 8, 9, 10 and 10.5 weeks of age (73 days of age), respectively, followed by challenge with the 9481 strain (8 × 10^9^ CFU) via drinking water at 11 weeks of age (77 days of age). The protection rates were 91.7% (22/24), 100% (24/24), 62.5% (15/24), 69.6% (16/23) and 83.3% (20/24), respectively, while the survival rate in the control group was 12.5% (3/24) [27]. This demonstrated that the CU strain could induce protective immunity against virulent *Pasteurella multocida* as early as 4 days post-vaccination in turkeys. It was hypothesized that the CU strain administered via WW puncture might occupy natural target sites in the host via certain mechanisms (analogous to the “occupancy effect” observed with pseudorabies vaccines [35]), thereby preventing wild-type pathogen invasion while simultaneously stimulating immune responses to further block infection. In the same year, Schlink et al. conducted similar experiments to evaluate the immunoprotective efficacy of the CU strain in turkeys under different physiological states (laying or producing semen). Results revealed that in the CU vaccinated group, four laying turkeys died (20/24 survived) with an overall 25% reduction in egg production, whereas all non-laying turkeys survived. After challenge with the 9481 strain (6 × 10^9^ CFU), both groups achieved comparable protection rates of 95% (19/20) and 96% (24/25), respectively. Under identical protocols, the semen producing group featured three deaths (20/23 survived) and five cases showing the clinical symptoms of “torticollis” while only two deaths were observed in the non-semen producing group. Both vaccinated groups achieved similar levels of protective efficacy upon challenge [31]. Although the protective efficacy of the CU strain did not differ significantly between non-laying/non-semen producing and laying/semen producing periods, turkeys producing eggs or semen exhibited higher susceptibility to the vaccine strain. Therefore, in vaccination programs, primary immunization should preferably be administered prior to the onset of egg production.

In summary, within a certain age range of chicks, the protective efficacy of drinking water vaccination with the CU strain increased with advancing age. During the growing period, immunoprotective efficacy against *Pasteurella multocida* could be induced as early as four days post-vaccination with the turkey CU strain. No significant difference in the immunoprotective efficacy was detected between non-laying/laying periods and semen/non-semen producing periods in turkeys, although during laying or semen production periods, turkeys exhibited greater sensitivity to the CU strain. Therefore, primary immunization should preferably be administered prior to the onset of laying periods (Table 3).

### 2.4. Immunization Efficacy of the CU Strain Vaccine in Various Animal Species

In 1982, Cai et al. demonstrated that the single-dose vaccination of 17 weeks of age adult ducks via drinking water or IM injection with the CU strain (1 × 10^9^ CFU/bird) resulted in 100% survival (4/4), with a protection rate of 2/4 against subsequent SC challenge with the N8112 strain (275 CFU/bird) at 18 days post-vaccination while all control ducks died (0/4). Under identical protocols, complete protection (4/4) was achieved in ducks receiving two-dose regimens (either dual IM injections or prime-drinking/boost-intramuscular administration) when challenged with 100 CFU/bird [29] (Table 4). These findings indicated that the CU strain exhibits favorable safety and protective efficacy in adult ducks via either drinking water or IM injection. Using comparable protocols, Cai et al. further evaluated the immunogenicity in other species. Two-dose vaccination of 54 weeks of age geese via oral gavage (3.8 × 10^9^, 1.9 × 10^9^, and 7.6 × 10^9^ CFU/bird) or SC injection (3.8 × 10^8^ and 2.1 × 10^8^ CFU/bird) with the CU strain yielded 100% survival, with survival rate ranging from 80% to 100% (4/5–5/5) against a challenge with the C48-1 strain at 24 days post-immunization, the survival rate of the control group ranging from 33.3% to 40% [29] (Table 4). Similarly, vaccinated 7 weeks of age goslings receiving oral (1.5 × 10^9^, 7.6 × 10^8^, and 3.8 × 10^8^ CFU/bird) or SC (8.5 × 10^7^ and 4.2 × 10^7^ CFU/bird) immunization showed 100% survival, with 75–100% protection (3/4–4/4) when challenged, compared to 40% survival (4/10) in the control group [29] (Table 4). In contrast, administration of the CU strain to 17 weeks of age rabbits via the oral (5.0 × 10^9^, 2.0 × 10^9^, 1.0 × 10^9^, 1.0 × 10^8^ and 1.0 × 10^7^ CFU/bird) or SC (2.6 × 10^9^, 1.3 × 10^9^, 1.0 × 10^8^, 5.0 × 10^5^ and 5.0 × 10^4^ CFU/bird) routes resulted in 100% mortality within three days for all SC groups, while oral groups exhibited survival rates of 0/4, 3/4, 3/4, 1/2 and 1/2, respectively. Remarkably, all surviving rabbits died to a challenge with the C51-2 strain (5 CFU/bird) at 24 days post-vaccination [29] (Table 4). These data collectively demonstrated that while the CU strain showed promising safety and partial protective efficacy in geese through both the oral and SC routes, it exhibited marked toxicity and failed to confer protective immunity in rabbits.

In 1996, Dabbert et al. investigated models of *Pasteurella multocida* infection in adult northern bobwhites (*Colinus virginianus*). The IM inoculation of 58,720 CFU or higher doses of the CU strain resulted in 100% mortality while doses of 2300 or 3000 CFU caused 30–75% mortality and doses of 230 CFU or lower did not induce mortality. Furthermore, single immunization with an aluminum hydroxide-adjuvanted inactivated CU strain vaccine (1.0 × 10^8^ CFU/bird) administered intramuscularly provided protection in 4/6 birds when challenged intramuscularly with the CU strain (1.2 × 10^4^ CFU) at 10 days post-vaccination compared to 2/6 survival in the control group. In contrast, two-dose immunization achieved 75% protection (15/20), while all birds from the control group died of infection (0/4) [36]. Collectively, these findings indicated that while the CU strain exhibited high virulence in northern bobwhites, its inactivated vaccine formulation achieved substantial homologous protective efficacy.

In conclusion, the CU strain has been demonstrated to exhibit favorable safety and protective efficacy in both vaccinated ducks and geese. However, this vaccine has been identified to possess significant virulence in northern bobwhites, while its inactivated vaccine formulation has been shown to provide effective homologous protection. Furthermore, oral or SC administration of the CU strain to domestic rabbits has been observed to result in partial or complete mortality, with subcutaneous inoculation demonstrating greater pathogenicity. Moreover, this vaccine did not confer protective immunity in immunized rabbits.

### 2.5. The Influence of Drugs on the Immunization Efficacy of the CU Strain Vaccine

In 1976, Bierer and Derieux divided a group of 10 weeks of age turkeys into five subgroups, which were inoculated with the CU strain via drinking water (30 mL culture added per 4000 mL of drinking water) and subsequently fed diets containing 0.1% sulfaquinoxaline starting on days 0, 1, 2, 3 and 4 post-inoculation, respectively. The medication regimen consisted of two days of feeding followed by three days of withdrawal, then two days of feeding at 0.05% concentration. At 14 days post-inoculation, challenge with the P-1059 strain (30 mL culture added per 4000 mL of drinking water for three consecutive days) resulted in protection rates of 2/10, 7/10, 8/10, 10/10 and 8/10, respectively, while the survival rates were 10/10 and 1/10 for the inoculated group which were fed a normal diet, and the blank control group, respectively [37]. These findings demonstrated the susceptibility of the CU strain to sulfaquinoxaline and indicated that the earlier administration of sulfonamides following CU strain inoculation in turkeys resulted in progressively lower immunoprotective efficacy. Therefore, the use of sulfonamide drugs should be avoided in animal populations during vaccination programs. In 1977, Olson et al. reported that feeding turkeys diets containing low concentrations of Rofenaid-40 (containing 0.01% potentiated sulfadimethoxine) before and after CU strain inoculation, as well as before and after challenge, had no significant effect on the immunoprotective efficacy of the CU strain or on the virulence of the 8579 strain [34] (Table 5), thus indicating the resistance of both *Pasteurella multocida* CU and 8579 strains to low concentrations of potentiated sulfonamides.

In 1976 and 1980, Derieux et al. conducted similar experimental studies which demonstrated that the inclusion of antimicrobial agents, such as furazolidone, bacitracin, arsanilic acid, oleandomycin, neomycin, sulfanitran, and dinsed in turkey diets before and after vaccination with the CU strain, or prior to challenge with either the P-1059 or X-73 strains, had no significant effect on the immunoprotective efficacy of the CU strain nor on the virulence of the P-1059 or X-73 strains. These findings revealed that *Pasteurella multocida* CU and X-73 strains exhibited certain resistance to these drugs. Furthermore, no significant effects were observed with eleven antiparasitic drugs or one iodine supplement (Table 5) [38,39]. However, among the antimicrobial agents tested, erythromycin demonstrated considerable impact, with a protection rate of 72.5% (29/40) in the immunized group receiving this treatment, compared to 97.5% (9/40) in the vaccinated group fed a standard diet [38,39]. In 1978, Dziuk et al. reported that the dietary inclusion of aflatoxin B-1 before and after vaccination with the CU strain had no significant effect on its immunoprotective efficacy in turkeys [40] (Table 5).

In 1987, Schlink et al. administered the immunosuppressant cyclophosphamide at high, medium, and low doses (16, 12, and 8 mg/bird, respectively) via intraperitoneal injection for three consecutive days to three groups of day-old Nicholas broad-breasted white turkeys, with mortality rates of 26/30, 21/30, and 8/30 observed in the respective groups. The surviving birds, along with a control group, were orally inoculated with the CU strain (4 × 10^8^ CFU) at seven weeks of age, resulting in mortality rates of 2/4, 0/9, 0/22, and 1/24 in the high-, medium-, and low-dose cyclophosphamide groups and the normal immunization group, respectively. At 11 weeks of age, the remaining birds were challenged orally with strain 9481 (6 × 10^9^ CFU), demonstrating protection rates of 100% (2/2), 22.2% (2/9), 45.5% (10/22), and 73.9% (17/23) in the high-, medium- and low-dose cyclophosphamide groups and the normal immunization group, respectively, with a survival rate of 3/24 in the blank control group [41] (Table 5). These results indicated that cyclophosphamide exhibited significant toxicity in young turkeys, with dose-dependent toxicity, and suppressed the immunoprotective efficacy of CU strain vaccination in a dose-dependent manner. In 1996, Dabbert et al. immunized two groups of adult northern bobwhites with a CU strain aluminum hydroxide-adjuvanted inactivated vaccine (1.0 × 10^8^ CFU/bird) via IM injection, with one group receiving concomitant dexamethasone injections at 0, 3, 6, and 9 days post-vaccination. Challenge with the CU strain via IM injection at 10 days post-vaccination yielded protection rates of 100% (6/6) and 50% (3/6) in the normal immunization and dexamethasone-treated groups, respectively [36] (Table 5), thus demonstrating that the anti-inflammatory drug dexamethasone suppressed the immunoprotective efficacy of CU strain vaccination in northern bobwhites.

In summary, the CU strain has been shown to be susceptible to antimicrobial agents such as sulfaquinoxaline and erythromycin, necessitating the avoidance of these drugs in animal populations during vaccination programs involving the CU strain. However, the CU strain exhibited certain resistance to sulfonamide potentiators (at low concentrations), furazolidone, bacitracin, arsanilic acid, neomycin, oleandomycin, sulfanitran, and ormetoprim. Furthermore, the CU strain demonstrated tolerance to various antiparasitic agents including Coyden, as well as to the iodine supplement ethylenediamine dihydroiodide and aflatoxin B-1, with no significant impact observed on immunoprotective efficacy when turkeys were fed diets containing these drugs or toxins. Furthermore, the immunosuppressant cyclophosphamide and the anti-inflammatory drug dexamethasone have been found to inhibit the immunoprotective efficacy following vaccination with the CU strain (Table 5).

### 2.6. Comparative Analysis of the Immunization Efficacy Between the CU Strain Vaccine and Other Vaccines

In 1972, Bierer et al. conducted a comparative vaccination study in turkeys (120 birds per group, two groups) using two immunization protocols: a live attenuated *Pasteurella multocida* CU strain vaccine (oral administration via drinking water at 1.4 × 10^8^ CFU/day at 12 weeks of age) and a trivalent oil-adjuvanted inactivated vaccine (containing serotype A:1 strain X-73, A:3 strain P-1059, and A:4 strain P-1662; 1 mL SC injection at 8 weeks of age). Of the turkeys vaccinated with the CU strain, infection was observed in five birds with one case of mortality. At 14 weeks of age, each group was divided into three subgroups (n = 40 per subgroup) and challenged with P-1059, P-1662, or X-73 strains via drinking water, respectively. Protection rates were determined as 38/39 versus 22/40, 36/36 versus 35/40, and 37/40 versus 32/40 for the live attenuated and inactivated vaccine groups, respectively. Prime-boost immunization (inactivated vaccine priming followed by a CU strain booster) significantly reduced CU strain-associated adverse effects (infection rate: 3/120 with one case of mortality) while achieving complete protection (39/39) against all three challenge strains [25]. These findings demonstrated that although the CU strain vaccine may induce vaccine-associated morbidity and mortality at specified doses, it provided a greater protective efficacy and broader cross-protection when compared to oil-adjuvanted inactivated vaccines. Furthermore, the prime-boost strategy effectively mitigated adverse reactions associated with the administration of live attenuated vaccines.

In 1989, Avakian et al. conducted a comparative study evaluating the immunoprotective efficacy of the live attenuated CU strain vaccine against commercial multivalent oil-adjuvanted inactivated vaccines (MBL and SAL) and two laboratory-prepared multivalent oil-adjuvanted inactivated vaccines (P-3 containing X-73, P-1059, and P-1662 strains; P-4 containing X-73, P-1059, P-1662, and CU strains). Results demonstrated that the CU strain vaccine exhibited a greater immunoprotective efficacy and a longer duration of immunity in broilers when compared to multivalent and oil-adjuvanted inactivated vaccines (P-3, P-4, commercial MBL and SAL). Furthermore, a prime-boost immunization regimen employing the CU strain (primary vaccination) followed by inactivated vaccine (secondary vaccination) was shown to induce stronger protective efficacy when compared to the double-dose administration of oil-adjuvanted inactivated vaccines [42] (Table 6).

In 1991, Friedlander et al. immunized three groups of 7 weeks of age female Nicholas broad-breasted white turkeys via drinking water with equal doses (5 × 10^8^ CFU/bird) of live attenuated *Pasteurella multocida* CU strain vaccine, M-9 strain (a chemically induced mutant of the CU strain; serotype A:3,4), and MN strain (a temperature-sensitive mutant). When challenged at 11 weeks of age with 7 × 10^9^ CFU of strain 9481 (serotype 3,4) via drinking water, the protection rates were 24/24, 15/24 and 10/24, respectively, while the survival rate of the control group was 2/24. Furthermore, turkeys vaccinated with different doses of the M-9 strain (5 × 10^9^ or 5 × 10^7^ CFU/bird) achieved protection rates of 23/24 and 9/24, respectively, when challenged with the 9481 strain, while the survival rate of the control group was 2:24. Furthermore, when the CU strain was administered at 7 and 8 weeks of age, complete protection (24/24) was achieved against identical challenge. Under similar conditions, the combined immunization with M-9/CU or MN/CU strains yielded protection rates of 21/24 and 23/24, respectively, while the survival rate of the control group was 2/24 [43]. Collectively, these results demonstrated that, at equal doses, the CU strain vaccine provided a significantly higher immunoprotection when compared to the M-9 and MN strains. While a 10-fold higher dose of the M-9 strain enhanced the protection observed, it remained weaker than the one conferred by the CU strain. Moreover, combined immunization with M-9 or MN strain plus the CU strain induced weaker protection when compared to two doses of the CU strain alone (Table 6). In 1997, Hopkins et al. conducted similar experiments comparing the CU strain with the temperature-sensitive mutant PM-1 strain, demonstrating comparable protective efficacy between the CU and PM-1 strains in vaccinated turkeys [44] (Table 6).

In summary, under specific vaccination conditions and dosages, the CU strain vaccine induced certain degrees of morbidity and even mortality in poultry. However, its overall protective efficacy in chickens was significantly higher to that of the oil-adjuvanted inactivated vaccine, demonstrating stronger cross-protective immunity and longer immune persistence. Furthermore, when administered in combination with inactivated vaccines, the CU attenuated vaccine was shown to induce enhanced levels of protective immunity. In addition, while the temperature-sensitive mutant PM-1 derived from the CU strain exhibited comparable immunoprotective efficacy to its parental CU strain, the chemically induced mutant M-9 demonstrated markedly reduced protective efficacy relative to the CU strain (Table 6).

## 3. Other Natural Attenuated Vaccines

In 1968, Bierer et al. isolated an attenuated serotype 3 strain of *Pasteurella multocida* from avian cholera cases. High-dose oral vaccination of 9 weeks of age turkeys with the serotype 3 strain (60 mL of culture in 3.785 L of water for 21 days) followed by challenge with a virulent *P. multocida* strain resulted in complete protection (30/30) compared to a survival rate of 13/30 in the control group. Under identical conditions, low-dose vaccination of 6 weeks of age turkeys (30 mL of culture in 3.785 L of water for 24 days) achieved a protection rate of 69/80 versus 18/78 in the control group [45]. These findings demonstrated that the natural attenuated serotype 3 strain provided effective immunoprotection in turkeys via continuous oral administration. In 1969, Bierer et al. further compared this serotype 3 attenuated strain with commercial oil-adjuvanted inactivated vaccines, confirming its greater protective efficacy when administered orally three (or six) times when compared to single (or double) SC injections of inactivated vaccines [46].

During the same year, Bierer et al. conducted similar experiments to compare the immunoprotective efficacy of a live attenuated serotype 2 *P. multocida* strain vaccine and its inactivated counterpart with five different commercial inactivated vaccines, including three oil-adjuvanted bacterins, one aluminum hydroxide gel-adjuvanted bacterin, and one aqueous-adjuvanted bacterin. Results demonstrated that the natural attenuated serotype 2 strain provided a certain protective immunity in turkeys and, when administered continuously via drinking water for five weeks, this strain had greater immunoprotective effects when compared to two SC inoculations with any of the five commercial inactivated vaccines [47]. However, both the serotype 2 natural attenuated strain and the serotype 3 natural attenuated strain lost their immunogenicity during laboratory passages and consequently were not widely adopted [33].

In 1979, Singer et al. isolated an attenuated serotype 3 *P. multocida* strain from healthy geese which was administered via aerosols to 28 day old Nicholas White turkeys at different doses (5 × 10^8^ and 5 × 10^7^ CFU/m^2^). When challenged at 49 days of age with a virulent serotype 3 strain (6 × 10^8^ CFU/m^2^), protection rates were 10/16 and 3/8, respectively. Similar results were obtained when challenged with the A:1 strain X-73. Further investigations by Singer et al. on the effect of vaccination frequency demonstrated that the single administration of this natural attenuated serotype 3 strain (5.0 × 10^9^ CFU/m^2^) provided moderate immunoprotection and strong cross-protection in turkeys, while multiple vaccinations enhanced protective efficacy [48]. While the CU strain induced protective immunity against virulent *P. multocida* as early as day 4 post-vaccination [27], the serotype 3 attenuated strain required a longer immunization period of 10–21 days. In another experiment, comparing the immunogenic efficacy of the serotype 3 strain with two other attenuated strains (type 2,5 strain 94 and type 3 strain 536), the natural attenuated serotype 3 vaccine strain exhibited a greater protection against challenge with virulent serotype 3 strains compared to both strain 94 and strain 536, but exhibited intermediate protection against A:1 strain X-73 challenge; this was stronger than strain 94 yet weaker than strain 536 [48].

In 2005, Myint et al. intranasally administered the natural attenuated *P. multocida* B:3,4 strain P4675 vaccine at 100-fold the recommended dose (2.0 × 10^9^ CFU/head) to 50 cattle and 39 buffaloes aged 6–12 months, with all vaccinated animals surviving, thus demonstrating the excellent safety profile of the P4675 strain in both cattle and buffaloes. In subsequent immunoprotection studies, Myint et al. inoculated seven buffaloes and eight cattle with the P4675 attenuated vaccine at the recommended intranasal dose (2.0 × 10^7^ CFU/head). Seven months post-vaccination, three buffaloes were challenged subcutaneously with the virulent B:2 strain (2.0 × 10^7^ CFU/head), achieving complete protection (3/3), Twelve months post-vaccination, the remaining four buffaloes and all eight cattle were challenged similarly, demonstrating protection rates of 3/4 and 8/8, respectively [49], thus indicating that the natural attenuated P4675 strain provided effective immunoprotection in both cattle and buffaloes. In a separate experiment, Myint et al. conducted passive immunization tests in mice using collected serum samples, revealing that the natural attenuated P4675 strain conferred cross-protective efficacy against challenges with E:2 and F:3,4 strains, while also exhibiting partial cross-protection against A:3,4 strain challenges [49].

In 1991, Lin et al. isolated the attenuated *P. multocida* HP strain from healthy swine, which was subsequently administered subcutaneously to groups of Hainan yellow chickens (two birds per group) at three different doses (5.3 × 10^8^, 1.1 × 10^8^, and 1.1 × 10^7^ CFU/bird). Complete survival was observed in all vaccinated groups. Three weeks post-vaccination, challenge with a virulent strain (74 CFU/bird, SC injection) resulted in complete protection across all immunized groups while (control group survival: 0/2) [50]. These findings indicated that the swine-origin attenuated *P. multocida* HP strain exhibited both safety and immunoprotective efficacy in chickens.

In 1996, Liu et al. isolated an attenuated strain R1-23 (5:A) from laboratory-preserved avian *P. multocida* cultures. When administered via drinking water at varying doses (1.7 × 10^11^–1 × 10^12^ CFU/bird) to susceptible purebred Langshan chickens (76 and 280 days old), Leghorns (180 days old), Lohmann chickens (90 days old), and Jingbai chickens (120 and 140 days old), complete survival was observed in all vaccinated groups [51], demonstrating the excellent safety profile of the R1-23 strain. In 1997, Liu et al. further evaluated the R1-23 live attenuated vaccine (2.5 × 10^9^ CFU/bird, drinking water administration) by challenging immunized chickens with the virulent C48-1 strain (4 × 10^7^ CFU/bird, intranasal inoculation) at different timepoints post-vaccination (1–6 months). Protection rates ranged from 76.7% to 88% (46/60–44/50), while control group survival rates remained at 0–4% (0/25–1/25) [52], thus confirming the strong immunoprotective efficacy and durable immunity conferred by the R1-23 strain.

Natural attenuated vaccine strains have been experimentally confirmed to exhibit favorable safety profiles and immunoprotective efficacy. Of these, the P4675 strain live vaccine has been widely utilized in the United Kingdom and Myanmar, whereas the natural attenuated of avian origin *P. multocida* strains of serotype 3 and 2 isolated by Bierer et al. were found to lose immunogenicity during laboratory passage and consequently failed to achieve widespread application [33]. Singer et al. conducted field trials with a natural attenuated *P. multocida* strain of serotype 3 and from goose origin, demonstrating satisfactory performance following vaccination in over 20,000 poultry. Currently, no commercially available natural attenuated vaccines exist in China, with the traditional attenuated G19E40 strain being predominantly employed for the prevention of avian pasteurellosis. Furthermore, in 2012, Long et al. isolated a thin-capsule *P. multocida* attenuated strain (PmCQ6) from pneumonic calf lung tissue [53]. Comparative analysis with the virulent strain PmCQ2 (LD50: 1 CFU) revealed that PmCQ6 exhibited significantly reduced virulence (LD50: 1.9 × 10^8^ CFU) and harbored a point mutation in the capsule synthesis-associated hyaC gene [53]. Collectively, these findings indicated the attenuated virulence of PmCQ6, although the comprehensive evaluation of its safety and protective efficacy requires further systematic investigation.

## 4. Conclusions and Future Perspectives

By undertaking a comprehensive review of the research history of natural attenuated *P. multocida* vaccines, it has been established that these vaccines exhibit distinct advantages in inducing cross-protective immunity and achieving a prolonged duration of immunity. Several successfully developed and widely used natural attenuated *P. multocida* strains, including CU and P4675, have been shown to possess excellent safety profiles and immunoprotective efficacy, and continue to play significant roles in the prevention and control of pasteurellosis in both livestock and poultry. In this review, the research history of natural attenuated *P. multocida* vaccines was systematically evaluated and summarized. First, the immunoprotective efficacy of the natural attenuated CU strain has been demonstrated to be influenced by multiple factors including dosage, administration route, target animal species, and the characteristics of challenge strains. When considering safety, immunoprotective effects, and operational feasibility in clinical practice, the optimal immunization methods have been identified as the administration via drinking water, SC injection, and WW puncture inoculation, these conventional approaches being particularly suitable for intensive farming systems. Furthermore, the attenuated CU strain exhibited broad-spectrum immunoprotection, providing effective protection in various avian species (turkeys, broilers, ducks, and geese), with overall protective efficacy significantly greater than commercial inactivated vaccines, demonstrating stronger cross-protection and longer-lasting immunity. Other natural attenuated *P. multocida* vaccine strains, such as P4675, HP, and R1-23, have also been confirmed to exhibit favorable safety and immunoprotective characteristics. Of these attenuated vaccines, CU strain vaccines have been widely adopted in North America and European countries under various brand names including COLERA VAC^®^, Orachol^®^, and Avichol, while live P4675 vaccines have been predominantly used in the United Kingdom and Myanmar. However, HP and R1-23 strains have yet to achieve widespread application. Currently, no commercial natural attenuated vaccines are available in China. Natural attenuated *P. multocida* vaccine strains have been found to exhibit sensitivity to certain antimicrobial agents, necessitating the avoidance of these drugs during vaccination programs. Therefore, systematic antimicrobial susceptibility testing should be conducted during the development of natural attenuated vaccine strains, with particular emphasis on evaluating the potential effects of medicated feeds containing these antimicrobials on the immunoprotective efficacy of *P. multocida* attenuated vaccines. Furthermore, immunosuppressive and anti-inflammatory drugs have been shown to potentially inhibit the immunoprotective effects following vaccination with naturally attenuated *P. multocida* vaccines. In addition, natural attenuated *P. multocida* vaccines have demonstrated considerable tolerance to various compounds including antiparasitic drugs, iodine supplements, and aflatoxins, with no significant adverse effects observed on immunoprotective efficacy when administered concurrently with feeds containing these substances or toxins [34,36,37,38,39,40,41].

Throughout the history of vaccine development, numerous natural attenuated vaccines, such as the Newcastle disease LaSota strain [16] and the avian pasteurellosis attenuated vaccine CU strain [17], have been widely used with demonstrated efficacy, significantly reducing both regional and global incidence and the mortality rates of infectious diseases [20,21,22]. The successful application of these vaccines has established promising prospects for the development of natural attenuated *P. multocida* vaccines. However, the identification of optimal natural attenuated vaccine strains requires extensive screening from natural sources, which is both time-consuming and labor-intensive, with inherent unpredictability. Furthermore, natural attenuated vaccine strains lack well-defined genetic backgrounds, particularly with regard to specific virulence-attenuating gene targets, making it impossible to monitor their dissemination within animal populations or assess potential virulence reversion risks. Currently, with advancements in genetic engineering and bioinformatics technologies, the introduction of genetic markers into natural attenuated strains and the establishment of detection methods to accurately differentiate between natural infection and vaccine immunization have become crucial for the control and prevention of *P. multocida* infections. Furthermore, comparative genomic analyses between virulent strains and natural attenuated strains can elucidate their genetic evolutionary mechanisms, thus enabling the identification of novel virulence genes or protective antigens. Collectively, these findings can subsequently facilitate the rapid construction of genetically engineered attenuated vaccine strains through homologous recombination or gene editing technologies targeting specific genes of interest.

## Figures and Tables

**Table 1:** Effects of vaccination routes on *P. multocida* CU strain immunogenicity.

Vaccination Route (Vaccination Frequency)	Animal Species	Vaccination Dose (CFU)	Challenge Strain	Serotype	Challenge Routes	Challenge Dose (CFU)	Survival Rate (No. Survived/Total)		References
							Vaccinated	Controls	
Drinking water	turkeys	7.5 × 10^6^	P-1059	A:3	DW	7.5 × 10^6^	100% (19/19)	30.0% (6/20)	[26]
web-wing puncture	turkeys	4.0 × 10^6^	P-1059	A:3	DW	7.5 × 10^6^	97.5% (39/40)	45.0% (18/40)	[26]
Intradermal	turkeys	2.0 × 10^7^	P-1059	A:3	DW	7.5 × 10^6^	85.0% (17/20)	30.0% (6/20)	[26]
Ocular	turkeys	3.4 × 10^7^	P-1059	A:3	DW	7.5 × 10^6^	83.3% (15/18)	30.0% (6/20)	[26]
Oral	turkeys	1.0 × 10^9^	P-1059	A:3	DW	7.5 × 10^6^	80.0% (16/20)	30.0% (6/20)	[26]
Feather follicle	turkeys	4.0 × 10^6^	P-1059	A:3	DW	7.5 × 10^6^	80.0% (8/10)	30.0% (6/20)	[26]
Palatine cleft	turkeys	2.5 × 10^8^	P-1059	A:3	DW	7.5 × 10^6^	70.0% (14/20)	30.0% (6/20)	[26]
Cloacal	turkeys	2.5 × 10^8^	P-1059	A:3	DW	7.5 × 10^6^	55.0% (11/20)	30.0% (6/20)	[26]
Nostril	turkeys	3.4 × 10^7^	P-1059	A:3	DW	7.5 × 10^6^	35.0% (7/20)	30.0% (6/20)	[26]
Drinking water	turkeys	4.0 × 10^8^	9481	3,4	DW	6.0 × 10^9^	70.8% (17/24)	20.8% (5/24)	[27]
Subcutaneous injection	turkeys	4.0 × 10^8^	9481	3,4	DW	6.0 × 10^9^	75.0% (18/24)	20.8% (5/24)	[27]
Intratracheal injection	turkeys	4.0 × 10^8^	9481	3,4	DW	6.0 × 10^9^	100% (23/23)	20.8% (5/24)	[27]
Bursal perfusion	turkeys	4.0 × 10^8^	9481	3,4	DW	6.0 × 10^9^	95.5% (21/22)	20.8% (5/24)	[27]
Wing-web puncture	turkeys	4.0 × 10^8^	9481	3,4	DW	6.0 × 10^9^	87.0% (20/23)	20.8% (5/24)	[27]
Crop injection	turkeys	4.0 × 10^8^	9481	3,4	DW	6.0 × 10^9^	82.6% (19/23)	20.8% (5/24)	[27]
Subcutaneous injection (2)	chickens	5.0 × 10^6^	X-73	A:1	PC	ND	100% (15/15)	3.85% (1/26)	[28]
Subcutaneous injection (3)	chickens	5.0 × 10^6^	X-73	A:1	PC	ND	100% (17/17)	14.7% (6/41)	[28]
Stick-wing (2)	chickens	5.0 × 10^6^	X-73	A:1	PC	ND	87.5% (35/40)	3.85% (1/26)	[28]
Stick-wing (3)	chickens	5.0 × 10^6^	X-73	A:1	PC	ND	94.4% (19/20)	14.7% (6/41)	[28]
Drinking water (3)	chickens	5.0 × 10^9^	C48-1	A:1	SC	10	20.0% (1/5)	0% (0/3)	[29]
Drinking water (3)	chickens	2.0 × 10^9^	C48-1	A:1	SC	10	40.0% (2/5)	0% (0/3)	[29]
Drinking water (3)	chickens	1.0 × 10^9^	C48-1	A:1	SC	10	60.0% (3/5)	0% (0/3)	[29]
Subcutaneous injection (2)	chickens	5.0 × 10^8^	C48-1	A:1	SC	10	100% (5/5)	0% (0/3)	[29]
Subcutaneous injection (2)	chickens	2.0 × 10^8^	C48-1	A:1	SC	10	100% (5/5)	0% (0/3)	[29]
Subcutaneous injection (2)	chickens	1.0 × 10^8^	C48-1	A:1	SC	10	100% (5/5)	0% (0/3)	[29]
DW-WW	turkeys	— ^a^	P-1059	A:3	PC	— ^c^	71.8% (51/71) ^d^	0% (0/58)	[30]
DW-WW (2)	turkeys	— ^a^	P-1059	A:3	PC	— ^c^	72.2% (52/72) ^d^	0% (0/56)	[30]
DW (3)-WW (2)	turkeys	— ^b^	P-1059	A:3	PC	— ^c^	80.0% (40/50) ^d^	0% (0/44)	[30]
Drinking water	turkeys	4.0 × 10^8^	9481	3,4	DW	6.0 × 10^9^	70.6% (12/17)	4.2% (1/24)	[31]
Drinking water (3)	turkeys	4.0 × 10^8^	9481	3,4	DW	6.0 × 10^9^	100% (23/23)	4.2% (1/24)	[31]
Drinking water (7)	turkeys	4.0 × 10^8^	9481	3,4	DW	6.0 × 10^9^	100% (24/24)	4.2% (1/24)	[31]
DW-WW-DW ^e^	turkeys	4.0 × 10^8^	9481	3,4	DW	6.0 × 10^9^	100% (25/25)	4.2% (1/24)	[31]
DW-WW (2) ^e^	turkeys	4.0 × 10^8^	9481	3,4	DW	6.0 × 10^9^	91.7% (22/24)	4.2% (1/24)	[31]
Auditory tube	turkeys	4.0 × 10^8^	9481	3,4	DW	6.0 × 10^9^	22.2% (2/9)	4.2% (1/24)	[31]
DW (2)-AT ^e^	turkeys	4.0 × 10^8^	9481	3,4	DW	6.0 × 10^9^	100% (24/24)	4.2% (1/24)	[31]
DW-AT-DW ^e^	turkeys	4.0 × 10^8^	9481	3,4	DW	6.0 × 10^9^	100% (23/23)	4.2% (1/24)	[31]
DW (3)-AT ^e^	turkeys	4.0 × 10^8^	9481	3,4	DW	6.0 × 10^9^	78.3% (18/23)	4.2% (1/24)	[31]
Total protective rate of two or more vaccinations via subcutaneous injection							100% (47/47)	9.2% (7/76)	
Total protective rate of two or more vaccinations via wing-web puncture							90.0% (54/60)	10.4% (7/67)	
Total protective rate of two or more vaccinations via drinking water							85.5% (53/62)	3.5% (2/57)	
Total protective rate of combined vaccinations via drinking water and wing-web puncture							78.5% (190/242)	1.0% (2/206)	
Total protective rate of combined vaccinations via drinking water and auditory tube							92.9% (65/70)	4.2% (3/72)	

DW = drinking water, PC = palatine cleft, SC = subcutaneous injection, WW = wing-web puncture, AT = auditory tube. ND, Not determined. ^a^ The drinking water dosage was 6.8 × 10^8^–7.9 × 10^8^ CFU per bird, the wing-web puncture vaccination dose was 7.5 × 10^7^ CFU per bird. ^b^ The drinking water dosage was 6.8 × 10^8^–7.9 × 10^8^ CFU per bird, the wing-web puncture vaccination dose was 7.0 × 10^7^–1.0 × 10^8^ per bird. ^c^ The challenge dose ranged from 2.0 × 10^8^ to 3.5 × 10^8^ CFU per bird. ^d^ Immunized animals were divided into six groups, with one group sequentially challenged every 5 weeks between 5 and 30 weeks post-final vaccination (total six challenge timepoints), from which the total protective rate was calculated. ^e^ All vaccination routes were administered at a standardized dose of 4.0 × 10^8^ CFU per bird.

**Table 2:** Effects of vaccination dosage and frequency on CU strain immunogenicity.

Vaccination Route (Vaccination Frequency)	Animal Species	Vaccination Dose (CFU)	Challenge Strain	Serotype	Challenge Routes	Challenge Dose (CFU)	Survival Rate (No. Survived/Total)		References
							Vaccinated	Controls	
Drinking water	turkeys	4.0 × 10^5^	P-1059	A:3	DW	2.0 × 10^7^	0% (0/30)	0% (12/12)	[32]
Drinking water	turkeys	6.0 × 10^7^	P-1059	A:3	DW	2.0 × 10^7^	76.7% (23/30)	0% (12/12)	[32]
Drinking water	turkeys	4.8 × 10^9^	P-1059	A:3	DW	2.0 × 10^7^	96.4% (27/28)	0% (12/12)	[32]
Drinking water (2)	broilers	1.5 × 10^8^/1.5 × 10^8^	P-1059	A:3	DW	2.0 × 10^7^	50.0% (10/20)	40.0% (8/20)	[33]
Drinking water (2)	broilers	1.4 × 10^8^/3.6 × 10^8^	P-1059	A:3	DW	2.0 × 10^7^	75.0% (30/40)	35.0% (14/40)	[33]
Drinking water (2)	broilers	1.5 × 10^8^/1.5 × 10^8^	X-73	A:1	DW	2.0 × 10^7^	25.0% (15/60)	28.3% (17/60)	[33]
Drinking water (2)	broilers	1.4 × 10^8^/3.6 × 10^8^	X-73	A:1	DW	2.0 × 10^7^	70.8% (34/48)	35.4% (17/48)	[33]
Drinking water	turkeys	2.0 × 10^9^	9481	3,4	DW	6.0 × 10^9^	100% (12/12)	25.0% (6/24)	[34]
Drinking water	turkeys	4.0 × 10^9^	9481	3,4	DW	6.0 × 10^9^	91.7% (11/12)	25.0% (6/24)	[34]
Drinking water	turkeys	8.0 × 10^9^	9481	3,4	DW	6.0 × 10^9^	100% (12/12)	25.0% (6/24)	[34]
Drinking water	turkeys	1.6 × 10^10^	9481	3,4	DW	6.0 × 10^9^	100% (12/12)	25.0% (6/24)	[34]
Wing-web puncture	turkeys	4.0 × 10^6^	9481	3,4	DW	6.0 × 10^9^	56.5% (13/23)	20.8% (5/24)	[27]
Wing-web puncture	turkeys	4.0 × 10^7^	9481	3,4	DW	6.0 × 10^9^	87.5% (21/24)	20.8% (5/24)	[27]
Wing-web puncture	turkeys	4.0 × 10^8^	9481	3,4	DW	6.0 × 10^9^	87.0% (20/23)	20.8% (5/24)	[27]
Wing-web puncture (1) ^a^	chickens	5.0 × 10^6^	X-73	A:1	PC	ND	77.8% (7/9) ^d^	3.85% (1/26)	[28]
Wing-web puncture (1) ^b^	chickens	5.0 × 10^6^	X-73	A:1	PC	ND	55.6% (5/9) ^d^	3.85% (1/26)	[28]
Wing-web puncture (2) ^ab^	chickens	5.0 × 10^6^	X-73	A:1	PC	ND	87.5% (35/40) ^d^	3.85% (1/26)	[28]
Wing-web puncture (1) ^a^	chickens	5.0 × 10^6^	X-73	A:1	PC	ND	45.5% (10/22) ^e^	14.6% (6/41)	[28]
Wing-web puncture (1) ^b^	chickens	5.0 × 10^6^	X-73	A:1	PC	ND	52.4% (11/21) ^e^	14.6% (6/41)	[28]
Wing-web puncture (2) ^ab^	chickens	5.0 × 10^6^	X-73	A:1	PC	ND	100% (20/20) ^e^	14.6% (6/41)	[28]
Wing-web puncture (3) ^a–c^	chickens	5.0 × 10^6^	X-73	A:1	PC	ND	95.0% (19/20) ^e^	14.6% (6/41)	[28]
Subcutaneous injection	turkeys	4.0 × 10^6^	9481	3,4	DW	6.0 × 10^9^	50.0% (12/24)	20.8% (5/24)	[27]
Subcutaneous injection	turkeys	4.0 × 10^7^	9481	3,4	DW	6.0 × 10^9^	33.3% (8/24)	20.8% (5/24)	[27]
Subcutaneous injection	turkeys	4.0 × 10^8^	9481	3,4	DW	6.0 × 10^9^	75.0% (18/24)	20.8% (5/24)	[27]
Subcutaneous injection	chickens	2.5 × 10^6^	C48-1	A:1	IM	14	100% (4/4)	33.3% (1/3)	[29]
Subcutaneous injection	chickens	5.0 × 10^6^	C48-1	A:1	IM	14	100% (4/4)	33.3% (1/3)	[29]
Subcutaneous injection	chickens	2.5 × 10^7^	C48-1	A:1	IM	14	75.0% (3/4)	33.3% (1/3)	[29]
Subcutaneous injection	chickens	6.7 × 10^7^	C48-1	A:1	IM	14	75.0% (3/4)	33.3% (1/3)	[29]
Drinking water	turkeys	4.0 × 10^8^	9481	3,4	DW	6.0 × 10^9^	70.6% (12/17)	4.2% (1/24)	[31]
Drinking water (3)	turkeys	4.0 × 10^8^	9481	3,4	DW	6.0 × 10^9^	100% (23/23)	4.2% (1/24)	[31]
Drinking water (7)	turkeys	4.0 × 10^8^	9481	3,4	DW	6.0 × 10^9^	100% (24/24)	4.2% (1/24)	[31]
DW-AT (2)	turkeys	4.0 × 10^8^/4.0 × 10^5^	9481	3,4	DW	6.0 × 10^9^	91.7% (11/12)	8.3% (1/12)	[31]
DW-AT (2)	turkeys	4.0 × 10^8^/4.0 × 10^6^	9481	3,4	DW	6.0 × 10^9^	91.7% (11/12)	8.3% (1/12)	[31]
DW-AT (2)	turkeys	4.0 × 10^8^/4.0 × 10^7^	9481	3,4	DW	6.0 × 10^9^	91.7% (11/12)	8.3% (1/12)	[31]
DW-AT (2)	turkeys	4.0 × 10^8^/4.0 × 10^8^	9481	3,4	DW	6.0 × 10^9^	75.0% (9/12)	8.3% (1/12)	[31]
Total protection rate of single vaccination via wing-web puncture							54.1% (33/61)	10.4% (14/134)	
Total protection rate of two-dose vaccination via wing-web puncture							91.7% (55/60)	10.4% (7/67)	
Total protection rate of three-dose vaccination via wing-web puncture							95.0% (19/20)	14.6% (6/41)	

DW = drinking water, PC = palatine cleft, IM = intramuscular injection, AT = auditory tube. ^a–c^ represent vaccination timepoints at 14 weeks-of-age, 22 weeks-of-age, 34 weeks-of-age, respectively. ^d,e^ represent challenge timepoints at 30 and 68 weeks-of-age, respectively. ND, Not determined.

**Table 3:** Effects of vaccination age on the immunogenic efficacy of the CU strain vaccine.

Vaccination Age (Days/Weeks)	Animal Species	Vaccination Routes	Vaccination Dose/CFU	Challenge Strain	Serotype	Challenge Age	Challenge Dose/CFU	Survival Rate (No. Survived/Total)		References
								Vaccinated	Controls	
1 d	turkeys	DW	2.0 × 107	P-1059	A:3	35 d	— ^a^	20.0% (2/10)	20.0% (4/20)	[17]
5 d	turkeys	DW	2.0 × 10^7^	P-1059	A:3	35 d	— ^a^	40.0% (4/10)	20.0% (4/20)	[17]
10 d	turkeys	DW	2.0 × 10^7^	P-1059	A:3	35 d	— ^a^	70.0% (7/10)	20.0% (4/20)	[17]
15 d	turkeys	DW	2.0 × 10^7^	P-1059	A:3	35 d	— ^a^	90% (9/10)	20.0% (4/20)	[17]
20 d	turkeys	DW	2.0 × 10^7^	P-1059	A:3	35 d	— ^a^	100% (10/10)	20.0% (4/20)	[17]
25 d	turkeys	DW	2.0 × 10^7^	P-1059	A:3	35 d	— ^a^	100% (10/10)	20.0% (4/20)	[17]
1 d	turkeys	DW	2.0 × 10^7^	P-1059	A:3	84 d	— ^a^	11.1% (1/9)	0% (0/19)	[17]
5 d	turkeys	DW	2.0 × 10^7^	P-1059	A:3	84 d	— ^a^	11.1% (1/9)	0% (0/19)	[17]
10 d	turkeys	DW	2.0 × 10^7^	P-1059	A:3	84 d	— ^a^	20.0% (2/10)	0% (0/19)	[17]
15 d	turkeys	DW	2.0 × 10^7^	P-1059	A:3	84 d	— ^a^	20.0% (2/10)	0% (0/19)	[17]
20 d	turkeys	DW	2.0 × 10^7^	P-1059	A:3	84 d	— ^a^	60.0% (6/10)	0% (0/19)	[17]
25 d	turkeys	DW	2.0 × 10^7^	P-1059	A:3	84 d	— ^a^	80.0% (8/10)	0% (0/19)	[17]
7 w	turkeys	WW	4.0 × 10^8^	9481	3,4	11 w	8.0 × 10^9^	91.7% (22/24)	12.5% (3/24)	[27]
8 w	turkeys	WW	4.0 × 10^8^	9481	3,4	11 w	8.0 × 10^9^	100% (24/24)	12.5% (3/24)	[27]
9 w	turkeys	WW	4.0 × 10^8^	9481	3,4	11 w	8.0 × 10^9^	62.5% (15/24)	12.5% (3/24)	[27]
10 w	turkeys	WW	4.0 × 10^8^	9481	3,4	11 w	8.0 × 10^9^	69.6% (16/23)	12.5% (3/24)	[27]
10.5 w (73 d)	turkeys	WW	4.0 × 10^8^	9481	3,4	11 w	8.0 × 10^9^	83.3% (20/24)	12.5% (3/24)	[27]
32 w (laying)	turkeys	DW	4.0 × 10^8^	9481	3,4	36 w	6.0 × 10^9^	95.0% (19/20)	8.3% (1/12)	[31]
32 w (nonlaying)	turkeys	DW	4.0 × 10^8^	9481	3,4	36 w	6.0 × 10^9^	96.0% (24/25)	8.3% (1/12)	[31]
32 w (SP)	turkeys	DW	4.0 × 10^8^	9481	3,4	36 w	6.0 × 10^9^	90.0% (18/20)	0% (0/5)	[31]
32 w (non-SP)	turkeys	DW	4.0 × 10^8^	9481	3,4	36 w	6.0 × 10^9^	95.5% (21/22)	0% (0/5)	[31]

DW = drinking water, WW = wing-web puncture, SP = semen producing, non-SP = non-semen producing. ^a^ Exposure, in each instance, was accomplished by the drinking water route, using a 1:50 dilution of a 20 h old brain-heart infusion broth culture as the only source of drinking water daily, for three consecutive days.

**Table 4:** Immunization efficacy of the CU strain vaccine in different animal species.

Vaccination Route (Vaccination Frequency)	Animal Species	Vaccination Dose (CFU)	Challenge Strain	Serotype	Challenge Route	Challenge Dose (CFU)	Survival Rate (No. Survived/Total)		References
							Vaccinated	Controls	
IM	adult ducks	1.0 × 10^9^	N8112	ND	IM	275	50.0% (2/4)	0% (0/4)	[29]
Oral	adult ducks	1.0 × 10^9^	N8112	ND	IM	275	50.0% (2/4)	0% (0/4)	[29]
IM (2)	adult ducks	1.0 × 10^9^/2.0 × 10^9^	N8112	ND	IM	100	100% (4/4)	0% (0/4)	[29]
Oral-IM	adult ducks	1.0 × 10^9^/2.0 × 10^9^	N8112	ND	IM	100	100% (4/4)	0% (0/4)	[29]
Oral (2)	adult geese	3.8 × 10^9^	C48-1	A:1	SC	6.0 × 10^3^	80.0% (4/5)	33.3% (1/3)	[29]
Oral (2)	adult geese	1.9 × 10^9^	C48-1	A:1	SC	6.0 × 10^3^	100% (5/5)	33.3% (1/3)	[29]
Oral (2)	adult geese	7.6 × 10^8^	C48-1	A:1	SC	6.0 × 10^3^	100% (5/5)	33.3% (1/3)	[29]
SC (2)	adult geese	3.8 × 10^8^	C48-1	A:1	SC	6.0 × 10^3^	100% (5/5)	33.3% (1/3)	[29]
SC (2)	adult geese	2.1 × 10^8^	C48-1	A:1	SC	6.0 × 10^3^	100% (5/5)	33.3% (1/3)	[29]
Oral (2)	goslings	1.5 × 10^9^	C48-1	A:1	SC	6.0 × 10^3^	80.0% (4/5)	40.0% (4/10)	[29]
Oral (2)	goslings	7.6 × 10^8^	C48-1	A:1	SC	6.0 × 10^3^	100% (4/4)	40.0% (4/10)	[29]
Oral (2)	goslings	3.8 × 10^8^	C48-1	A:1	SC	6.0 × 10^3^	80.0% (4/5)	40.0% (4/10)	[29]
SC (2)	goslings	8.5 × 10^7^	C48-1	A:1	SC	6.0 × 10^3^	100% (4/4)	40.0% (4/10)	[29]
SC (2)	goslings	4.2 × 10^7^	C48-1	A:1	SC	6.0 × 10^3^	75.0% (3/4)	40.0% (4/10)	[29]
Oral	adult rabbits	2.0 × 10^9^	C51-2	A:1	SC	5	0% (0/3)	— ^a^	[29]
Oral	adult rabbits	1.0 × 10^9^	C51-2	A:1	SC	5	0% (0/3)	— ^a^	[29]
Oral	adult rabbits	1.0 × 10^8^	C51-2	A:1	SC	5	0% (0/1)	— ^a^	[29]
Oral	adult rabbits	1.0 × 10^7^	C51-2	A:1	SC	5	0% (0/1)	— ^a^	[29]
IM	northern bobwhites	1.0 × 10^8^	CU	A:3,4	IM	1.2 × 10^4^	66.7% (4/6)	33.3% (2/6)	[36]
IM (2)	northern bobwhites	1.0 × 10^8^	CU	A:3,4	IM	1.2 × 10^4^	75% (15/20)	0% (0/4)	[36]
Total protection rate in twice-vaccinated adult ducks							100% (8/8)	0% (0/4)	
Total protection rate in twice-vaccinated adult geese							96.0% (24/25)	0% (0/8)	
Total protection rate in twice-vaccinated goslings							86.3% (19/22)	33.3% (5/15)	
Total protection rate in single-vaccinated adult rabbits							0% (0/8)	40% (20/50)	
Total protection rate in twice-vaccinated *Colinus virginianus*							75% (15/20)	— ^a^	

IM = intramuscular injection, SC = subcutaneous injection. ^a^ No parallel control group was included in this experiment. ND, Not determined.

**Table 5:** Effects of drug interference on the immunogenicity of the CU strain vaccine.

Vaccination Age (Days/Weeks)	Animal Species	Drug	Drug Added/Withdrawn Time	Challenge Time	Challenge Strain	Serotype	Survival Rate (No. Survived/Total)		References
							Vaccinated	Controls	
10 w	turkeys	Sulfaquinoxaline	70 d/76 d	12 w	P-1059	A:3	20.0% (2/10)	10.0% (1/10)	[37]
10 w	turkeys	Sulfaquinoxaline	71 d/77 d	12 w	P-1059	A:3	70.0% (7/10)	10.0% (1/10)	[37]
10 w	turkeys	Sulfaquinoxaline	72 d/78 d	12 w	P-1059	A:3	80.0% (8/10)	10.0% (1/10)	[37]
10 w	turkeys	Sulfaquinoxaline	73 d/79 d	12 w	P-1059	A:3	100% (10/10)	10.0% (1/10)	[37]
10 w	turkeys	Sulfaquinoxaline	74 d/80 d	12 w	P-1059	A:3	80.0% (8/10)	10.0% (1/10)	[37]
10 w	turkeys	/	/	12 w	P-1059	A:3	100% (10/10)	10.0% (1/10)	[37]
6 w	turkeys	Rofenaid-40 ^d^	5 w/12 w	10 w	8579	ND	95.8% (23/24)	33.3% (8/24)	[34]
6 w	turkeys	Rofenaid-40 ^d^	5 w/12 w ^a^	10 w	8579	ND	95.8% (23/24)	33.3% (8/24)	[34]
6 w	turkeys	/	/	10 w	8579	ND	100% (24/24)	33.3% (8/24)	[34]
35 d	turkeys	0.011% Furazolidone	21 d/33 d	47 d	P-1059	A:3	90.0% (27/30)	0% (0/30)	[38]
35 d	turkeys	0.011% Furazolidone	21 d/32 d	47 d	P-1059	A:3	83.3% (25/30)	0% (0/30)	[38]
35 d	turkeys	0.011% Furazolidone	21 d/31 d	47 d	P-1059	A:3	83.3% (25/30)	0% (0/30)	[38]
35 d	turkeys	0.011% Furazolidone	21 d/30 d	47 d	P-1059	A:3	93.3% (28/30)	0% (0/30)	[38]
35 d	turkeys	0.011% Furazolidone	21 d/29 d	47 d	P-1059	A:3	86.7% (26/30)	0% (0/30)	[38]
35 d	turkeys	0.011% Furazolidone	21 d/28 d	47 d	P-1059	A:3	80.0% (24/30)	0% (0/30)	[38]
35 d	turkeys	0.011% Furazolidone	31 d/46 d	47 d	P-1059	A:3	95.0% (38/40)	20.0% (8/40)	[38]
35 d	turkeys	0.011% Furazolidone	36 d/46 d	47 d	P-1059	A:3	95.0% (38/40)	20.0% (8/40)	[38]
35 d	turkeys	0.011% Furazolidone	38 d/46 d	47 d	P-1059	A:3	95.0% (38/40)	20.0% (8/40)	[38]
35 d	turkeys	0.011% Furazolidone	40 d/46 d	47 d	P-1059	A:3	90.0% (36/40)	20.0% (8/40)	[38]
/	turkeys	0.011% Furazolidone	31 d/61 d	47 d	P-1059	A:3	35.0% (7/20)	20.0% (8/40)	[38]
35 d	turkeys	/	/	49 d	X-73	A:1	97.5% (39/40)	7.5% (3/40)	[39]
35 d	turkeys	0.025% Coyden ^d^	28 d/48 d	49 d	X-73	A:1	97.5% (39/40)	7.5% (3/40)	[39]
35 d	turkeys	0.01875% Zoamix ^d^	28 d/48 d	49 d	X-73	A:1	97.5% (39/40)	7.5% (3/40)	[39]
35 d	turkeys	0.025% Ipropran	28 d/48 d	49 d	X-73	A:1	95.0% (38/40)	7.5% (3/40)	[39]
35 d	turkeys	0.0375% Carbosep	28 d/48 d	49 d	X-73	A:1	95.0% (38/40)	7.5% (3/40)	[39]
35 d	turkeys	Bacitracin	28 d/48 d	49 d	X-73	A:1	92.5% (37/40)	7.5% (3/40)	[39]
35 d	turkeys	0.01% Arsanilic acid	28 d/48 d	49 d	X-73	A:1	90.0% (36/40)	7.5% (3/40)	[39]
35 d	turkeys	Oleandomycin	28 d/48 d	49 d	X-73	A:1	87.5% (35/40)	7.5% (3/40)	[39]
35 d	turkeys	Neomycin	28 d/48 d	49 d	X-73	A:1	87.5% (35/40)	7.5% (3/40)	[39]
35 d	turkeys	Erythromycin ^d^	28 d/48 d	49 d	X-73	A:1	72.5% (29/40)	7.5% (3/40)	[39]
35 d	turkeys	/	/	/	X-73	A:1	95.0% (38/40)	0% (0/40)	[39]
35 d	turkeys	0.025% Amprolium ^d^	28 d/48 d	49 d	X-73	A:1	95.0% (38/40)	0% (0/40)	[39]
35 d	turkeys	0.01875% Nitarsone	28 d/48 d	49 d	X-73	A:1	92.5% (37/40)	0% (0/40)	[39]
35 d	turkeys	0.005% Roxarsone	28 d/48 d	49 d	X-73	A:1	90.0% (36/40)	0% (0/40)	[39]
35 d	turkeys	0.0375% Butynorate ^d^	28 d/48 d	49 d	X-73	A:1	87.5% (35/40)	0% (0/40)	[39]
35 d	turkeys	EDDI ^d^	28 d/48 d	49 d	X-73	A:1	85.0% (34/40)	0% (0/40)	[39]
35 d	turkeys	0.08% Dimetridazole	28 d/48 d	49 d	X-73	A:1	85.0% (34/40)	0% (0/40)	[39]
40 d	turkeys	/	33 d/47 d	54 d	P-1059	A:3	45.0% (9/20)	10.0% (1/10)	[39]
40 d	turkeys	Sulfanitran-Butynorate	33 d/47 d	54 d	P-1059	A:3	60.0% (6/10)	10.0% (1/10)	[39]
40 d	turkeys	Dinsed-Roxarsone	33 d/47 d	54 d	P-1059	A:3	50.0% (5/10)	10.0% (1/10)	[39]
56 d	turkeys	/	/	70 d	P-1059	A:3	100% (5/5)	40.0% (2/5)	[40]
56 d	turkeys	Aflatoxin B-l	49 d/70 d	70 d	P-1059	A:3	100% (5/5)	40.0% (2/5)	[40]
/	turkeys	Aflatoxin B-l	49 d/70 d	70 d	P-1059	A:3	0% (0/5)	40.0% (2/5)	[40]
56 d	turkeys	/	/	84 d	P-1059	A:3	100% (5/5)	40.0% (2/5)	[40]
56 d	turkeys	Aflatoxin B-l	49 d/70 d	84 d	P-1059	A:3	100% (5/5)	40.0% (2/5)	[40]
/	turkeys	Aflatoxin B-l	49 d/70 d	84 d	P-1059	A:3	40.0% (2/5)	40.0% (2/5)	[40]
56 d	turkeys	/	/	112 d	P-1059	A:3	100% (4/4)	40.0% (2/5)	[40]
56 d	turkeys	Aflatoxin B-l	49 d/70 d	112 d	P-1059	A:3	80.0% (4/5)	40.0% (2/5)	[40]
/	turkeys	Aflatoxin B-l	49 d/70 d	112 d	P-1059	A:3	20.0% (1/5)	40.0% (2/5)	[40]
7 w	turkeys	/	/	11 w	9481	3,4	73.9% (17/23)	12.5% (3/24)	[41]
7 w	turkeys	12 mg CTX	0 d/3 d	11 w	9481	3,4	22.2% (2/9)	12.5% (3/24)	[41]
7 w	turkeys	16 mg CTX	0 d/3 d	11 w	9481	3,4	100% (2/2)	12.5% (3/24)	[41]
7 w	turkeys	8 mg CTX	0 d/3 d	11 w	9481	3,4	45.5% (10/22)	12.5% (3/24)	[41]
— ^b^	NB	/	/	— ^c^	CU	A:3,4	100% (6/6)	33.3% (2/6)	[36]
— ^b^	NB	1 mg Dexamethasone	— ^b^	— ^c^	CU	A:3,4	50.0% (3/6)	33.3% (2/6)	[36]
Total Protection rate of vaccinated groups fed standard diet							97.5% (115/118)	19.3% (23/119)	
Total protection rate of vaccinated groups fed Coyden-supplemented diet							97.5% (39/40)	7.5% (3/40)	
Total protection rate of vaccinated groups fed Zoamix-supplemented diet							97.5% (39/40)	7.5% (3/40)	
Total protection rate of vaccinated groups fed Rofenaid-40-supplemented diet							95.8% (46/48)	33.3% (8/24)	
Total protection rate of vaccinated groups fed Amprolium-supplemented diet							95.0% (38/40)	0% (0/40)	
Total protection rate of vaccinated groups fed Butynorate-supplemented diet							87.5% (35/40)	0% (0/40)	
Total protection rate of vaccinated groups fed Ethylenediamine dihydroiodide-supplemented diet							85.0% (34/40)	0% (0/40)	
Total protection rate of vaccinated groups fed Erythromycin-supplemented diet							72.5% (29/40)	7.5% (3/40)	

EDDI = Ethylenediamine dihydroiodide, CTX = Cyclophosphamide, NB = Northern bobwhites. “/” indicates the control group without drug administration. ^a^ Animals in the experimental group were supplemented with Rofenaid-40 (containing 0.01% potentiated sulfadimethoxine) in their diet from 5 to 12 weeks-of-age, with treatment interruption from two days before vaccination until three days post-vaccination. ^b^ Vaccinated animals were adult northern bobwhites (*Colinus virginianus*), administered dexamethasone (0.5 mL/bird) via intramuscular injection at 0, 3, 6 and 9 days post-vaccination (dpv). ^c^ Challenge was performed at 10 days post-immunization (dpi). ^d^ This drug remains in clinical use, while other agents have been replaced in many countries due to safety concerns (toxicity, drug residues, antimicrobial resistance), with some regions imposing complete bans. ND, Not determined.

**Table 6:** Protective efficacy of the CU strain vaccine when compared with other vaccines.

Vaccine	Vaccination Route	Vaccination Age (Weeks)	Challenge Strain/Serotype	Challenge Route	Challenge Time	Survival Rate (No. Survived/Total)		References
						Vaccinated	Controls	
CU strain	DW	8 w	P-1059/A:3	PC	14 w	97.5% (38/39)	0% (0/4)	[25]
Trivalent inactivated vaccine	SC	12 w	P-1059/A:3	PC	14 w	55.0% (22/40)	0% (0/4)	[25]
Trivalent inactivated vaccine/CU strain	SC/DW	8/12 w	P-1059/A:3	PC	14 w	100% (39/39)	0% (0/4)	[25]
CU strain	DW	8 w	P-1662/A:4	PC	14 w	100% (36/36)	20.0% (8/40)	[25]
Trivalent inactivated vaccine	SC	12 w	P-1662/A:4	PC	14 w	87.5% (35/40)	20.0% (8/40)	[25]
Trivalent inactivated vaccine/CU strain	SC/DW	8/12 w	P-1662/A:4	PC	14 w	100% (39/39)	20.0% (8/40)	[25]
CU strain	DW	8 w	X-73/A:1	PC	14 w	92.5% (37/40)	25.0% (10/40)	[25]
Trivalent inactivated vaccine	SC	12 w	X-73/A:1	PC	14 w	80.0% (32/40)	25.0% (10/40)	[25]
Trivalent inactivated vaccine/CU strain	SC/DW	8/12 w	X-73/A:1	PC	14 w	100% (39/39)	25.0% (10/40)	[25]
CU strain	WW	12/21 w	X-73/A:1	PC	42 w	100% (10/10)	0% (0/25)	[42]
Multivalent inactivated P-4 vaccine	SC	12/21 w	X-73/A:1	PC	42 w	90.0% (18/20)	0% (0/25)	[42]
Commercial inactivated MBL vaccine	SC	12/21 w	X-73/A:1	PC	42 w	88.9% (8/9)	0% (0/25)	[42]
Commercial inactivated SAL vaccine	SC	12/21 w	X-73/A:1	PC	42 w	88.9% (8/9)	0% (0/25)	[42]
Multivalent inactivated P-3 vaccine	SC	12/21 w	X-73/A:1	PC	42 w	80.0% (16/20)	0% (0/25)	[42]
CU strain/MBL	SC	12/21 w	X-73/A:1	PC	42 w	100% (10/10)	0% (0/25)	[42]
CU strain/SAL	SC	12/21 w	X-73/A:1	PC	42 w	100% (10/10)	0% (0/25)	[42]
CU strain	WW	12/21 w	X-73/A:1	PC	72 w	88.5% (23/26)	2.9% (1/34)	[42]
Multivalent inactivated P-4 vaccine	SC	12/21 w	X-73/A:1	PC	72 w	71.4% (25/35)	2.9% (1/34)	[42]
Multivalent inactivated P-3 vaccine	SC	12/21 w	X-73/A:1	PC	72 w	60.5% (23/38)	2.9% (1/34)	[42]
Commercial inactivated MBL vaccine	SC	12/21 w	X-73/A:1	PC	72 w	57.1% (4/7)	2.9% (1/34)	[42]
Commercial inactivated SAL vaccine	SC	12/21 w	X-73/A:1	PC	72 w	42.9% (3/7)	2.9% (1/34)	[42]
CU strain/MBL	SC	12/21 w	X-73/A:1	PC	72 w	100% (10/10)	2.9% (1/34)	[42]
CU strain/SAL	SC	12/21 w	X-73/A:1	PC	72 w	60.0% (6/10)	2.9% (1/34)	[42]
CU strain	DW	7 W	9481/3,4	DW	11 w	100% (24/24)	8.3% (2/24)	[43]
M-9 strain	DW	7 W	9481/3,4	DW	11 w	62.5% (15/24)	8.3% (2/24)	[43]
MN strain	DW	7 W	9481/3,4	DW	11 w	41.7% (10/24)	8.3% (2/24)	[43]
M-9 strain/CU strain	DW	7/8 W	9481/3,4	DW	11 w	87.5% (21/24)	8.3% (2/24)	[43]
MN strain/CU strain	DW	7/8 W	9481/3,4	DW	11 w	95.8% (23/24)	8.3% (2/24)	[43]
CU strain/CU strain	SC/DW	7/8 W	9481/3,4	DW	11 w	100% (24/24)	8.3% (2/24)	[43]
CU strain ^b^	DW	7 w	9481/3,4	DW	11 w	61.74% (29/47)	15.8% (9/57)	[44]
PM-1 strain ^b^	DW	7 w	9481/3,4	DW	11 w	62.5% (30/48)	15.8% (9/57)	[44]
Total protection rate in CU strain-vaccinated groups						88.7% (197/222)	13.4% (30/224)	
Total protection rate in commercial inactivated vaccine-vaccinated groups ^a^						73.7% (112/152)	9.9% (20/202)	
Total protection rate in P-4 inactivated vaccine-vaccinated groups						78.2% (43/55)	1.7% (1/59)	
Total protection rate in P-3 inactivated vaccine-vaccinated groups						67.2% (38/58)	1.7% (1/59)	
Total protection rate in M-9 strain-vaccinated groups						62.5% (15/24)	8.3% (2/24)	
Total protection rate in MN strain-vaccinated groups						41.7% (10/24)	8.3% (2/24)	
Total protection rate in PM-1 strain-vaccinated groups ^b^						62.5% (30/48)	15.8% (9/57)	

DW = drinking water, PC = palatine cleft, SC = subcutaneous injection, WW = wing-web puncture. ^a^ the total protection rates in three commercial inactivated vaccine groups (trivalent inactivated vaccine, MBL strain inactivated vaccine, and SAL strain inactivated vaccine). ^b^ Under identical experimental conditions, the PM-1 strain demonstrated comparable immunoprotective efficacy to the CU strain.

## Data Availability

No new data were created or analyzed in this study.

## Abbreviations

The following abbreviations are used in this manuscript:

Pm	*Pasteurella multocida*
DW	Drinking water
WW	wing-web puncture
SC	subcutaneous injection
PC	Palatine cleft
AT	Auditory Tube
IM	Intramuscular injection
